# Analytical performances and field assessment of a multiplex Real-Time PCR assay for the simultaneous detection of *Echinococcus multilocularis* and *Echinococcus granulosus* sensu stricto in wild canids fecal samples

**DOI:** 10.1371/journal.pone.0362682

**Published:** 2026-11-12

**Authors:** Graziana Da Rold, Filippo Marzoli, Federica Obber, Roberto Celva, Alessia Franzoso, Giacomo De Conti, Elisa Dalla Libera, Patrizia Danesi, Sofia Sgubin, Rudi Cassini, Erica Marchiori, Dominik Trenkwalder, Piero Bonelli, Giovanna Masala, Chiara Rossi, Heidi Christine Hauffe, Carlo Vittorio Citterio

**Affiliations:** 1 Department of Animal Medicine, Production and Health, University of Padova, Legnaro, Italy; 2 Istituto Zooprofilattico Sperimentale delle Venezie (IZSVe), Legnaro, Italy; 3 Wildlife Management Office, Autonomous Province of Bolzano/Bozen – South Tyrol, Italy; 4 National Reference Center for Echinococcosis (CeNRE), Istituto Zooprofilattico Sperimentale della Sardegna “G. Pegreffi”, Sassari, Italy; 5 Conservation Genomics Research Unit, Centre for Research and Innovation, Fondazione Edmund Mach (FEM), San Michele all’ Adige, Italy; Xinjiang Medical University Affiliated First Hospital, CHINA

## Abstract

*Echinococcus multilocularis* and *Echinococcus granulosus sensu lato* are the causative agents of alveolar and cystic echinococcosis, two important zoonoses with significant public health implications. Several diagnostic approaches are currently available for the detection of these parasites in definitive hosts, including classical parasitological methods and molecular techniques. However, conventional techniques such as intestinal scraping or copromicroscopy are often labor-intensive, time-consuming, and require skilled personnel, while some molecular assays may show limited sensitivity or specificity. This study aimed at developing and assessing a real-time PCR assay using species-specific probes for the diagnosis of *E. multilocularis* and *E. granulosus sensu stricto* directly from the feces of potential definitive hosts, i.e., domestic and wild canids. The methodological assessment was performed by estimating the Limit of Detection (LoD), and specificity and the assay was applied to the analysis of fresh, field-collected canid fecal samples. Our protocol demonstrated excellent analytical performance, achieving an LoD corresponding to a single egg per reaction (from 0.10 g of feces) and a specificity of 100% for both *Echinococcus* species. Moreover, by relying directly on DNA extraction from fecal material and eliminating the need for sequencing through the use of specific probes, this method significantly reduced laboratory time and associated costs. The assay was successfully applied to field samples collected during surveillance activities, providing results consistent with the known biology and epidemiology of *Echinococcus* species in the study area.

## 1. Introduction

Echinococcosis is caused by tapeworms of the genus *Echinococcus*, affecting both humans and animals. This disease group includes alveolar echinococcosis (AE) and cystic echinococcosis (CE), caused by *E. multilocularis* (Leuckart 1863) and *E. granulosus* (Batsch, 1786) sensu lato (s.l.) respectively [1], which represent the second and the third most relevant food-borne zoonotic parasitic diseases in the world [2,3]. Humans can contract the infection by accidental ingestion of *Echinococcus* eggs acting as epidemiological dead-end hosts. Human CE primarily affects the liver and lungs but its mortality is low, in contrast to AE that is often fatal in untreated cases, due to tumor-like, infiltrative, and metastatic growth [1,4–7]. Echinococcosis remains a significant public health threat in Europe, where a declining trend in traditionally endemic CE regions is currently contrasted by a rising incidence of AE across the continent [6–9]. *E. multilocularis* is widespread among Central Europe, with red foxes (*Vulpes vulpes*) serving as the primary definitive host. Their high population density and susceptibility result in substantial worm burdens and prevalence rates reaching up to 30–50% in highly endemic areas [10–12], with increases expected in northern EU and the Alps under current climate scenarios [13]. Wild canids like red foxes, wolves (*Canis lupus*) and golden jackals (*Canis aureus*) become infected by predation of intermediate rodent hosts, like mice and voles, carrying the parasite. Despite their susceptibility to infection, domestic dogs contribute relatively little to environmental contamination in Europe due to their low exposure to infected rodents and the widespread practice of periodic deworming [2,10]. *E. multilocularis* is well-established and reported in many European areas: Switzerland, eastern France (with recent expansions toward the south-east), southern Germany, Austria, northern Scandinavia and the Balkans [7]. In Italy, the parasite is present in the Trentino-Alto Adige/Südtirol Region [14] although recent evidence suggests the presence of this cestode in other areas, such as the western Italian alps [15] and the Apennines [16]. Notably, in Italy, the year 2023 marked the confirmation of the first autochthonous human AE case in the endemic province of Alto Adige/Südtirol [17]. Although *E. granulosus* s.l. was previously considered a single species, it is now recognized as an assemblage of cryptic species with different host specificities, as well as varying human pathogenicity and infectivity. *E. granulosus* s.l. is currently subdivided into *E. granulosus* sensu stricto (s.s.) (with genotypic variants G1-G3), *Echinococcus felidis*, *Echinococcus equinus* (G4), *Echinococcus ortleppi* (G5) and *Echinococcus canadensis* (G6/G7, G8, G10) [18]. The domestic dog is the primary definitive host driving the synanthropic cycle of *E. granulosus* s.s., while wild canids (e.g., wolves, golden jackals) are predominantly involved in the epidemiology of other *Echinococcus* species (e.g., *E. multilocularis*) or specific sylvatic genotypes within the complex such as *E. canadensis* G8 and G10. However, they may also play a secondary or marginal role in maintaining sylvatic cycles of *E. granulosus* s.s. in certain regions, such as parts of Southern/Eastern Europe, Australia, sub-Saharan Africa, and sub-Arctic transition zones [6,19–21].

Sheep and goats are, specifically, the most important domestic intermediate hosts for *E. granulosus* s.s [22–24]., while a wider range of domestic livestock hosts, including cattle, yak, buffalo, camelids, pigs and equids, are susceptible intermediate hosts to *E. granulosus* s.l. and/or involved in the transmission of this tapeworm [25]. Northeastern Italy is considered a hypo-endemic area for CE. Autochthonous foci in bovines were identified in the Veneto Region through a retrospective and spatial analysis in 2006–2010 [26]. The main source of environmental contamination is represented potentially by sheep dogs that move extensively with their flocks throughout northeastern Italy, during the traditional transhumant system [27]. Indeed, *E. granulosus* s.s. is the species suspected to circulate in this area, since it accounts for the vast majority of *Echinococcus* infections in southern Europe [12,28]. Furthermore, a research study performed on slaughtered sheep evidenced the presence of *E. granulosus* s.s. genotypes G1 and G3 in North East Italy (Emilia Romagna, Veneto and Trentino Alto Adige) [29].

In the European Union (EU), the surveillance and control of *Echinococcus* spp. in pets, livestock, and wildlife are strictly regulated. Under Directive EU 2003/99 (monitoring of zoonoses) and Regulation EU 2016/429 (Animal Health Law), reporting detections in both humans and potentially susceptible animal species to national authorities is mandatory, while the collection of comprehensive metadata including epidemiological distribution, prevalence, and wildlife population data is highly recommended [30,31]. Given these public health mandates, combined with the fragmented and changing distributional patterns of these parasites (particularly *E. multilocularis*) [7,32], the deployment of rapid and highly sensitive diagnostic tools is crucial.

To date, the methods used for the diagnosis of *Echinococcus* infections in definitive and intermediate animal hosts include classical and biomolecular parasitological methods. Among the former, in definitive hosts, techniques such as examination of adult worms from intestinal scrapings of carcasses using stereomicroscopy, and copromicroscopic examination of parasite eggs in fresh fecal samples [33–37] have been commonly used. However, copromicroscopy does not permit species differentiation, whereas intestinal scraping requires access to intestinal tissue, typically from necropsied animals. This requirement changes the sampling approach and limits the availability of suitable samples for large-scale surveillance, while also making the procedure more time-consuming. Since the pioneering diagnostic applications of PCR in the 1990s for the coprological detection and discrimination of *Echinococcus* species [38–40], molecular techniques have rapidly evolved. Over the past decades, molecular techniques such as conventional polymerase chain reaction (PCR) [41–43] and real-time PCR [44–49] have been developed as rapid and reliable diagnostic tools, offering high suitability for species and genotype discrimination. A combined diagnostic approach integrating traditional and molecular techniques has also been proposed, namely copromicroscopy followed by multiplex PCR (CMPCR) and sequencing for cestode species identification [14]. Although this strategy was designed to optimize resources by limiting molecular analyses to copromicroscopy-positive samples, it demonstrated a very low diagnostic sensitivity compared to the real-time PCR assay developed by Obber et al. [50].

The aim of the present study was to set up and assess the diagnostic characteristics of a rapid and sensitive multiplex one-step TaqMan real-time PCR assay targeting *E. multilocularis* and *E. granulosus* s.s. in fecal samples. Lastly, to validate the protocol and assess its applicability to field collected samples, fecal samples collected from carcasses of wild canids within a surveillance activity ongoing in north-eastern Italy between 2024 and 2025, were analyzed using the newly developed method.

## 2. Materials and methods

### 2.1 Reference material and samples

The reference material used for *E. multilocularis* consisted of adult worms collected from the intestines of red foxes culled in the Autonomous Province of Bolzano/Bozen – South Tyrol from 2019 to 2020 and feces of red foxes sampled from carcasses in Veneto Region and the Province of Trento from 2012 to 2021 and stored at IZSVe laboratories. Adult worms were recovered from intestines using the scraping, filtration and counting technique (SFCT) according to Gesy et al. [51]. Gravid proglottids were dissected under a stereomicroscope to isolate a known number of eggs, which were subsequently used to spike red fox fecal samples previously confirmed negative for *Echinococcus* through host intestinal scraping as well as real-time PCR and multiplex endpoint PCR for cestodes, according to the diagnostic flow and protocols validated as described by Citterio et al. [14] and Knapp et al. [52]. Additionally, field-collected fecal samples confirmed negative for the target pathogens via real-time PCR and multiplex endpoint PCR for cestodes were used to evaluate the matrix effect and assess DNA degradation in environmental samples. These *Echinococcus*-negative red fox fecal (ENFF) samples served as a realistic field-relevant matrix.

*Echinococcus granulosus* s.s. DNA extracted from two positive dog fecal samples, confirmed by real-time PCR and sequencing by the Istituto Zooprofilattico Sperimentale della Sardegna “G. Pegreffi,” National Reference Centre for Echinococcosis (IZS-Sardegna CeNRE) were used as reference material for *E. granulosus* s.s. Furthermore, domestic dog fecal samples, confirmed negative for *E. granulosus* s.s. via both endpoint and real-time PCR were extracted to obtain clean matrix DNA, which was subsequently used for serial dilutions of the positive reference DNA.

In each real-time PCR run, a confirmed ENFF sample was used as a negative process control (NPC). Positive template controls (PTC) consisted of DNA from adult *E. multilocularis* (19/135414) specimens and *E. granulosus* s.s. cysts (TV-176/22), ensuring consistent target detection and assay performance.

### 2.2 Test of analytical sensitivity

The analytical sensitivity for *E. multilocularis* was assessed in four replicates, using a known quantity of eggs (300 per replicate) isolated from gravid proglottids. Nine 1:2 serial dilutions in Dulbecco phosphate buffered saline (PBS-D) were prepared and 1 mL of each dilution was used to spike 0.10 g of ENFF sample (1:10 dilution). DNA was subsequently extracted from spiked samples and stored at −20°C until use.

The analytical sensitivity for *E. granulosus* s.s. was assessed in two replicates, using DNA extracted from positive dog feces (see Section 2.1), due to the absence of adult specimens and/or eggs. This DNA was added to DNA extracted from ENFF and subjected to nine 1:2 serial dilutions. The use of DNA extracted from ENFF was needed to consider the presence of potential inhibitors in fecal material. The resulting dilutions were analyzed in duplicate, as the quantity of available material was limited. The highest dilution yielding at least two positive PCR replicates was considered to be the Limit of Detection (LoD) of the method for both *E. multilocularis* and *E. granulosus* s.s [53].

### 2.3 DNA extraction from feces

This step was performed for ENFF spiked with *E. multilocularis* eggs and ENFF. *E. multilocularis* samples were prepared in 2 mL tubes, as described in Section 2.2, and a 5 mm steel bead was added to each tube to ensure egg lysis. For ENFF samples, an aliquot ranging from 0.10 g to 0.17 g was weighed into a 2 mL tube, a 5 mm steel bead was added, and the sample was diluted 1:10 in PBS-D. All samples were homogenized using TissueLyser (RRID:SCR_025420) at 30 Hz for 5 minutes and then centrifuged at 1,000 g for 1 minute. For each sample, 200 μL of the supernatant and 9 μL of QuantiNova Internal Control (IC) DNA (Qiagen) were transferred into a 96-deep-well DNA extraction plate. DNA extraction was performed using MagMAX™ Pathogen RNA/DNA Kit (Thermofisher Scientific, Massachusetts, USA) and the Hamilton Microlab STAR liquid handler (RRID:SCR_019993), according to the manufacturers’ protocol. Elution was performed in 90 µl of the Elution buffer and stored at −20°C until use.

### 2.4 Real-time PCR conditions and correlation of number of eggs and cycle threshold

A multiplex real-time PCR assay was used to detect *E. multilocularis* and *E. granulosus* s.s. Primers and specific probes sequences Em-rrn F, Em-rrn R, Probe EM, for *E. multilocularis* [52] and Eg-COXF, Eg-COXR, Eg_COXP, for *E. granulosus sensu stricto* [46] are shown in Table 1. DNA amplification was performed using the QuantiNova Pathogen +IC Kit (QIAGEN, Hilden, Germany). The PCR assay was carried out in a final volume of 20 μl containing 5.6 μl of nuclease free water, 5 μl of QuantiNova Pathogen Master Mix (1X), 1 μl of primer Em-rrn F (0.5 μM), 1 μl of primer Em-rrn R (0.5 μM), 1 μl of primer Eg-COXF (0.5 μM), 1 μl Eg-COXR primer (0.5 μM), 0.2 μl of Probe EM (0.1 μM), 0.2 μl of Eg_COX_P (0.1 μM), 2 μl of QuantiNova IC Assay 1X and 3 μl of extracted DNA. Real-Time PCR was performed using the CFX96 Touch Real-Time PCR System (RRID:SCR_018064) and QuantStudio™ 5 Real-Time PCR System (RRID:SCR_020240) under the following condition: 95°C for 2 min, 40 cycles of 95°C for 5s, and 60°C for 30s. Fluorescence was measured at the end of each cycle. Signals for *E. multilocularis, E. granulosus* s.s. and the IC were detected in the FAM, Cy5 and HEX channels, respectively. A negative extraction control (NPC) consisting of PBS-D and IC and a PCR “no template control” (NTC; nuclease free water) were included in all real-time PCR runs. Positive controls consisted of DNA extracted from adult specimens of *E. multilocularis* and DNA extracted from *E. granulosus* s.s. cysts.

**Table 1:** Primers, probes and internal control employed for the detection of *E. multilocularis* and *E. granulosus* s.s. by multiplex real-time PCR. The table includes forward and reverse primers, TaqMan probes, their respective fluorescent dyes and the QuantiNova internal control used in the assay.

Target	Gene	Oligo name	Sequence (5'- 3')
*E. multilocularis*		Em-rrn F	5’-CTGTGATCTTGGTGT AGTAGTTGAGATTT-3’
	*rrnL*	Em-rrn R	5’-GGCTTACGCCGGTCTTAACTC -3’
		Probe EM	FAM-TGGTCTGTTCGACCTTTTTAGCCTCCAT-TAMRA
*E. granulosus* s.s.		Eg-COXF	5’-AGGGGCTGGTGTTGGTTGGA-3’
	COX1	Eg-COXR	5’-TGAAACACCAGCCAAATGCAGAGA-3’
		Eg_COX_P	CY5-TCCGCCGTTGTCCTCGTCGT-BHQ2
Internal Control (IC)		QuantiNova IC	HEX-labelled; sequence not disclosed by the manufacturer

To correlate Cycle threshold (Ct) values with egg counts, a calibration curve was constructed using the *E. multilocularis* real-time PCR results, where serial dilutions represented known numbers of eggs. Data were analyzed using GraphPad Prism software by plotting the Log10 transformed egg counts (X-axis) against the corresponding Ct values (Y-axis). While this curve provides a biological reference, the assay is intended for qualitative use; therefore, any quantitative interpretation is purely indicative.

### 2.5 Test of analytical specificity and repeatability

The principles of exclusivity (exclusion of false-positives) and inclusivity (correct identification of the target DNA) have been investigated [54] to evaluate the ability of the method to differentiate *Echinococcus* from most common species of the genus *Taenia*, and to identify the DNA of the two species of *Echinococcus*. Furthermore, a specific “Cross-Detection Trial” was performed to evaluate the method’s ability to successfully differentiate *E. multilocularis* from *E. granulosus* s.s., ensuring the simultaneous identification of both species in the same sample.

To evaluate exclusivity, DNA from non-target species (primarily other taeniids) was employed. The Parasitology Laboratory of Istituto Zooprofilattico Sperimentale delle Venezie (IZSVe) provided DNA extracted from fox feces previously screened and confirmed positive for cestodes other than *Echinococcus*. These samples were characterized using multiplex endpoint PCR and subsequent sequencing [14] to ensure definitive species identification. Specifically, the DNA from the following tapeworms was used: *Taenia krabbei* (PAR-1706/18)*, Dipylidium caninum* (PAR-827/17)*, Taenia polyacantha* (PAR-6761/19)*, Taenia crassiceps* (PAR-492/20), *Hydatigera taeniaeformis* (PAR-4629/2/20) and *Taenia hydatigena* (PAR-987/19). In addition, bacterial DNA extracted from a reference strain of *Escherichia coli* ATCC 25922 (TV-646/2/06) was tested. All samples were subjected to the multiplex real-time PCR in a single replica. To ensure result reliability, positive and negative controls were included in each plate. DNA extracted from fecal samples previously identified as positive for *E. multilocularis* (BL-371/20, BL-2753/19, BL-384/20, PAR-6157/18, BL-1707/19) or *E. granulosus* s.s. (TV-118C/22, TV-160C/22) by multiplex endpoint PCR was amplified for inclusivity tests.

Additionally, within the Cross-Detection Trial, nine simulated mixed samples (Sample 1 to Sample 9) were prepared, using a cross-titration matrix by combining DNA dilutions of both species (1:2 [low], 1:16 [medium], and 1:256 [high]), in all reciprocal combinations (Table 2). All samples were subjected to the multiplex real-time PCR in a single replica to assess assay performance in cases of target co-presence, particularly under highly asymmetric ratios where target competition or amplification bias might occur.

**Table 2:** Experimental layout of the cross-titration matrix used for the multiplex real-time PCR Cross-Detection Trial. Nine simulated co-infection mixtures (Sample 1 to Sample 9) were generated by cross-combining three scalar DNA dilutions, Low (1:2), Medium (1:16), and High (1:256) of *E. multilocularis* and *E. granulosus* s.s. The 3 X 3 grid includes both balanced ratios (Samples 1, 5, and 9) and asymmetric ratios (Samples 2, 3, 4, 6, 7, and 8) to evaluate assay efficiency, potential target competition, and preferential amplification during co-detection.

		*E. multilocularis*		
***E. granulosus* s.s.**	**Dilutions**	Low (1:2)	Medium (1:16)	High (1:256)
	Low (1:2)	Sample 1	Sample 2	Sample 3
	Medium (1:16)	Sample 4	Sample 5	Sample 6
	High (1:256)	Sample 7	Sample 8	Sample 9

The intra-laboratory precision of the method was assessed by performing repeated tests under repeatability conditions, evaluating both within-run and between-day variance across two different operators. For each target pathogen, three serial dilutions (low [1:2], medium [1:16], and high [1:256]) were amplified in triplicate by each operator within the same analytical run and across three independent testing days. Repeatability was evaluated based on the percentage of overall result agreement, with an acceptance threshold defined as at least 95% concordant outcomes.

### 2.6 Field assessment using samples from surveillance of wild carnivores

The newly developed method was assessed through the analysis of wild canid fecal samples collected in 2024–2025, according to an ongoing surveillance activity. Red foxes legally hunted during hunting season or found dead from the territory of South Tyrol (province endemic for *E. multilocularis*), and wolves found dead in the whole territory of northeastern Italy were submitted to the IZSVe. At necropsy, fecal samples were collected directly from the rectum, using appropriate personal protective equipment (PPE) and frozen at −80˚C for at least 72h to inactivate *Echinococcus* eggs. Each sample was subsequently georeferenced using a WebGIS developed by the IZSVe for the spatial localization and visualization of red foxes and other wild canids. All fecal samples were then tested using the described method.

### 2.7. Ethics statement

Formal ethical approval was not required for this study because the fox specimens were obtained from carcasses of animals found dead or legally culled under the official wildlife population management plan of the Autonomous Province of Bolzano/Bozen – South Tyrol, and no animals were killed specifically for this research. Fecal samples were collected non-invasively from animals in natural settings. Informed consent was not applicable because the study did not involve live animals, animal experimentation, or interventions on animals.

## 3. Results

### 3.1 Analytical sensitivity

The results of the sensitivity testing for *E. multilocularis* and *E. granulosus* s.s. are summarized in Table 3 and the raw data are reported in S1 and S2 Tables. Amplification was detected up to the eighth dilution (1:256), with Ct values ranging from 38.11 to 38.98. This dilution is estimated to correspond approximately to a single *E. multilocularis* egg, and therefore represents the limit of detection (LoD) of the method. Based on these results, a Ct threshold of 38.00 was established to define a sample as positive.

**Table 3:** Analytical sensitivity of the multiplex real-time PCR assay for *E. multilocularis* and *E. granulosus* s.s. Results of the analytical sensitivity assessment for *E. multilocularis* and *E. granulosus* s.s. For *E. multilocularis*, negative red fox feces were spiked with known numbers of eggs. For each dilution step, the table reports the number of spiked eggs, the corresponding Log_10_ transformed egg count, and the mean cycle threshold (Ct) value. For *E. granulosus* s.s., serial dilutions of DNA were prepared in DNA extracted from negative dog feces; the corresponding mean Ct values are reported for each dilution. The 1:1024 dilution was assessed only for *E. granulosus* s.s., NA (not applicable), dashes (--) denote unamplified samples.

	*E. multilocularis*			*E. granulosus* s.s.
Samples	N. of eggs	Log_10_ (N. of eggs)	Mean Ct value	Mean Ct value
**Full concentration**	300	2.48	30.14	30.46
**Dilution 1:2**	150	2.18	31.08	31.33
**Dilution 1:4**	75	1.88	32.42	32.17
**Dilution 1:8**	37.5	1.57	33.34	33.31
**Dilution 1:16**	18.8	1.27	34.30	34.24
**Dilution 1:32**	9.4	0.97	35.07	35.12
**Dilution 1:64**	4.7	0.67	36.10	36.25
**Dilution 1:128**	2.3	0.36	37.46	37.47
**Dilution 1:256**	1.2	0.08	38.40	38.50
**Dilution 1:512**	0.6	--	--	39.31
**Dilution 1:1024** ^**a**^	NA	NA	NA	--

^a^The 1:1024 dilution was assessed only for *E. granulosus* s.s., NA (not applicable)

The average Ct value of the four replicates obtained during the sensitivity testing for *E. multilocularis* were associated to the Log_10_ of the number of eggs to generate a standard curve correlating the number of eggs with the corresponding Ct values (Fig 1), yielding an R² value of 0.9972 and an amplification efficiency of 97.3%.

**Fig 1:**
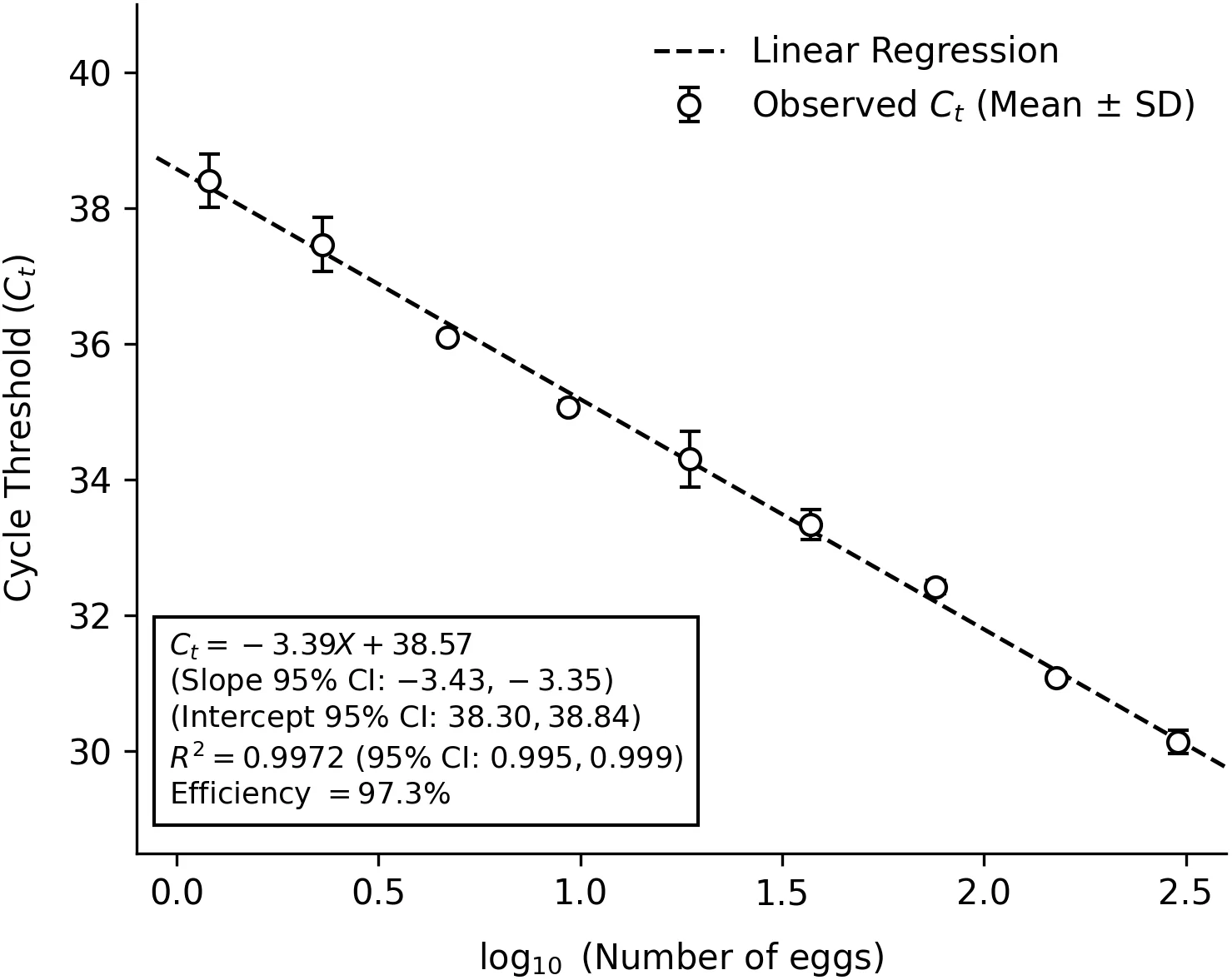
Scatter plot of number of eggs and cycle threshold (Ct). The standard curve is generated using a Log_10_ serial dilution of egg points ranging from 1.2 to 300 eggs per reaction (n = 4 replicates per dilution point, total n = 36). Circles represent the observed mean cycle threshold (Ct) values, with error bars indicating the standard deviation (SD) of four operational replicates per point to visualize technical assay variability. The dashed line denotes the linear regression trend (Ct = −3.39X + 38.57). Inset box reports comprehensive regression statistics, including 95% confidence intervals (CI) for the slope (95% CI: −3.43 to −3.35) and Y-intercept (95% CI: 95% CI: 38.30 to 38.84), coefficient of determination (R^2^ = 0.9972, 95% CI: 0.995, 0.999) and amplification efficiency (E = 97.3%).

The results of the sensitivity testing for *E. granulosus* s.s. are shown in S2 Table. All dilutions were successfully amplified. As shown in S2 Table, the analytical LoD for *E. granulosus* s.s. reached the ninth dilution (1:512), with Ct values ranging from 39.20 to 39.42. This high sensitivity is attributed to the use of purified DNA for the serial dilutions rather than DNA extracted directly from the fecal matrix. Based on the standard curve generated using known numbers of *E. multilocularis* eggs (Fig 1), the threshold of Ct ≤ 38.00 corresponding to approximately one egg, was established to define a positive sample, and this threshold was also applied to *E. granulosus* s.s. Although the assay detected *E. granulosus* s.s. DNA at lower concentrations (Ct ~ 39), the Ct ≤ 38.00 threshold was uniformly applied to both species. This direct comparison and uniform cut-off are methodologically justified by the comparable amplification kinetics and efficiencies of the two targets within the multiplex assay, as confirmed by the absence of competition or systematic Ct shifts in cross-detection trials (S3 Table). Because the primers and probes for *E. multilocularis* and *E. granulosus* s.s. possess highly comparable amplification efficiencies, their Ct values can be analytically compared to standardize the diagnostic threshold across both pathogens based on a shared baseline. Notably, this uniform cut-off is not mere extrapolation of detectable egg equivalents, but it is supported by identical Limit of Detection (LoD) behavior in the fecal matrix. By spiking purified *E. granulosus* s.s. DNA into negative fecal DNA, we demonstrated that matrix effects and LoD remain equivalent whether starting from feces naturally contaminated with eggs or from DNA diluted in a fecal background. However, while analytically valid to standardize the diagnostic cut-off, these Ct values cannot be used to quantify absolute egg burdens in natural, field-collected mixed fecal samples, where the assay remains strictly qualitative. Fluorescence signals for the IC consistently appeared at a Ct of ~27.3, indicating the absence of PCR inhibitors commonly found in feces and confirming reliable DNA extraction and amplification across all samples.

### 3.2 Analytical specificity and repeatability

The primers and probes used in the present method showed high specificity for the genus *Echinococcus*. The exclusivity of the method is illustrated in Fig 2 by the presence of two amplification curves corresponding to the positive controls of *E. granulosus* s.s. and *E. multilocularis* which are amplified at low cycle numbers, with Ct values of Ct = 27.76 and Ct = 18.91, respectively. No amplification was observed in samples positive for other tapeworm species and *E. coli* (see S4 Table for details), confirming the absence of cross reactivity (Fig 2).

**Fig 2:**
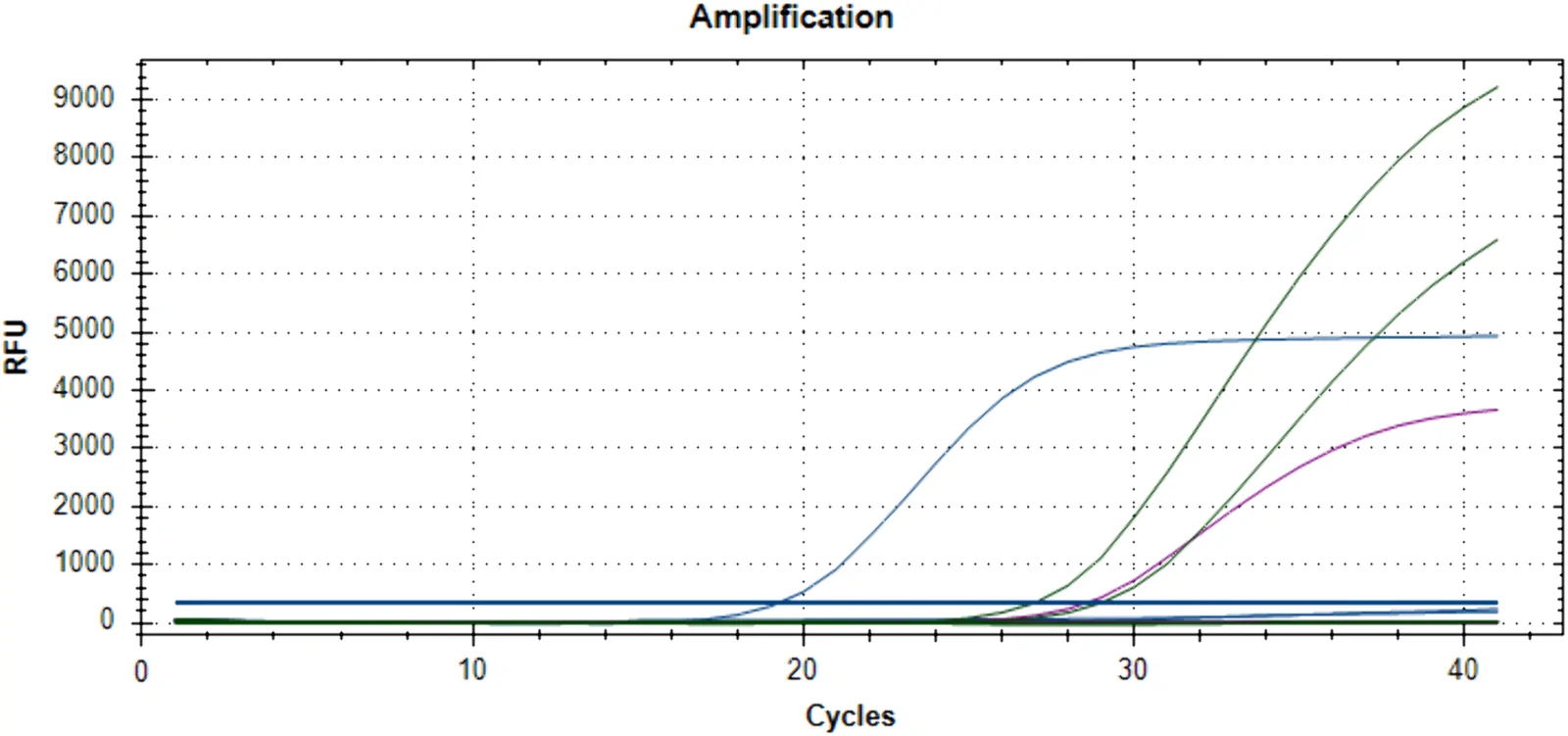
Real-time multiplex PCR amplification plot. Amplification curves generated with Design & Analysis Software v2.6 (Applied Biosystems). The graph displays Relative Fluorescence Units (RFU) versus PCR cycle number (Cycles). The blue curve corresponds to *E. multilocularis* DNA amplification, red curve represents *E. granulosus* s.s. DNA amplification, and green curves depict the internal controls (IC) amplification. The colored horizontal lines indicate the respective fluorescence threshold values set for target detection. DNA samples from non-target pathogens (1 bacterium, 1 *Dypilidium caninum* and 5 species of *Taenia*) show no fluorescence increase above the baseline, demonstrating the high analytical specificity of the primer-probe sets for the targets.

Additional results from the Cross-Detection Trial, conducted using mixtures of DNA from *E. multilocularis* and *E. granulosus* s.s. at low (1:2), medium (1:16), and high (1:256) dilutions to assess assay specificity, are provided in S3 Table. Overall, amplification signals were detected for both targets in all combinations, with FAM and Cy5 fluorophores specifically associated with *E. multilocularis* and *E. granulosus* s.s., respectively. This simultaneous detection without apparent interference demonstrates that the multiplex assay effectively identifies both species in mixed samples. As expected, Ct values increased with higher dilutions (lower DNA concentration), confirming the analytical performance of the amplification system.

The multiplex real-time PCR assay exhibited high intra-laboratory repeatability and technical precision across testing days and operators. Raw data of intra-laboratory repeatability are reported in S5 and S6 Tables. Overall, the method showed a qualitative repeatability rate, expressed as the percentage of overall result agreement, of 100% for *E. multilocularis* and 98% for *E. granulosus* s.s., confirming high analytical reliability across all tested contamination levels. Analysis of Ct values for *E. multilocularis* contaminated feces demonstrated remarkable consistency across all serial dilutions (low [1:2], medium [1:16], and high [1:256]). Across two operators and three independent testing days (18 replicates per dilution), the overall mean Ct values (± SD) were 30.05 ± 0.27 for the high contamination level (1:2), 32.89 ± 0.44 for the medium level (1:16), and 36.91 ± 0.58 for the low level (1:256). The intra-laboratory coefficient of variation (%CV) remained consistently low across all operational conditions: 0.90% for high (1:2), 1.34% for medium (1:16), and 1.58% for low (1:256) contamination levels (S5 Table). Regarding *E. granulosus* s.s., analysis of Ct values demonstrated high technical precision across all concentration levels: at the high contamination level (1:2), 17 out of 18 replicates were successfully amplified, yielding a mean Ct value of 30.54 ± 1.12 (%CV = 3.66%). Only a single replicate on Day 3 (Operator 1) yielded an unamplified result (ND). At the medium contamination level (1:16), 100% of replicates (18/18) were detected with a mean Ct of 34.69 ± 0.25 and an exceptionally low %CV of 0.71%. At the low contamination level (1:256), 100% of replicates (18/18) were likewise detected with a mean Ct of 37.40 ± 0.33 (%CV = 0.89%) (S6 Table). Intra- and inter-assay precision between operators and across independent runs was high, with consistently low SD (range:0.27–0.58 for *E. multilocularis*; 0.25–1.12 for *E. granulosus* s.s.) and %CV values (range: 0.90%–1.58% for *E. multilocularis*; 0.71%–3.66% for *E. granulosus* s.s.) demonstrating the robust analytical performance of the qualitative assay for both target species.

### 3.3. Field assessment

Our new protocol was applied to canid fecal samples of unknown infection status, collected in the framework of ongoing surveillance. A total of 250 samples from red foxes (n = 168) and wolves (n = 82) were collected from carcasses and analysed in the period 2024–2025. The number of samples tested for species and results for *E. multilocularis* and *E. granulosus* s.s. are reported in Table 4. The raw data for all samples, including the results of the real-time PCR assays, are provided in S7 Table. Overall, *E. multilocularis* was detected only in red foxes, whereas *E. granulosus* s.s. was detected only in wolves. The geographical distribution of tested and positive samples is shown in Fig 3.

**Table 4:** Detection of *E. multilocularis* and *E. granulosus* s.s. in wild canid samples. Screening and positivity results obtained using the novel real-time PCR protocol.

		*E. multilocularis*		*E. granulosus* s.s.	
Host species	Total fecal samples tested	N. positive samples	Positivity (%)	N. positive samples	Positivity (%)
*C. lupus* (grey wolf)	82	0	0%	4	4.9%
*V. vulpes* (red fox)	168	8	4.8%	0	0%

**Fig 3:**
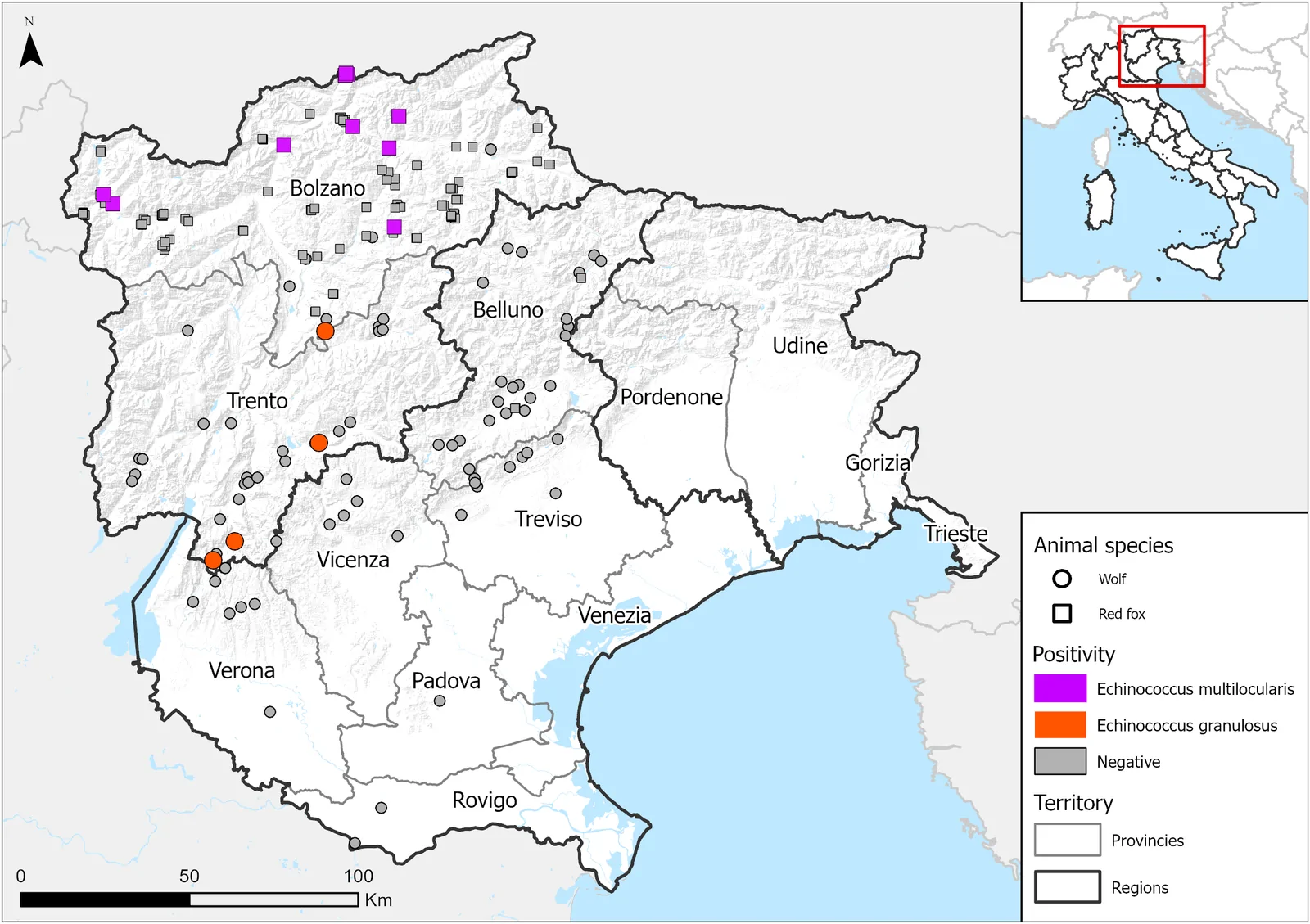
Field distribution of positive wild canid fecal samples. Grey wolf samples are indicated by outlined circles and red fox samples by outlined squares. Violet, orange, and grey denote *E. multilocularis* positive, *E. granulosus* s.s. positive, and negative samples, respectively.

## 4. Discussion

Infections caused by *E. multilocularis* and *E. granulosus* s.s. represent significant zoonotic threats, highlighting the need for sensitive, rapid, multi-target, and high-throughput diagnostic approaches for pathogen identification. Effective monitoring of these parasites requires the analysis of large, representative sets of animal samples, making rapid and reliable diagnostic methods critically important. In this study, we developed and assessed a real-time multiplex PCR assay capable of simultaneously detecting *E. multilocularis* and *E. granulosus* s.s. directly from fecal samples of domestic and wild canids. Although this study tested the assay on intestinal contents from red foxes and wolves, the method holds potential for broader application to feces from other wild and domestic canids and potentially felids, which may be sampled during field surveillance. This technique represents a significant diagnostic advancement over the approach proposed by Obber et al. [50], as it enables the simultaneous detection of both major zoonotic cestodes. Developing a real-time PCR capable of simultaneously detecting *E. multilocularis* and *E. granulosus* s.s. is essential in regions where the distribution of the two parasites overlaps. However, it should be noted that co-infections in individual host animals are rare in nature due to differences in host preferences and ecological cycles (e.g., lower prevalence of *E. multilocularis* in domestic dogs, and minimal involvement of foxes for *E. granulosus* s.s.). Furthermore, detection from fecal samples is particularly advantageous, as samples can be collected in the field using standardized protocols, processed rapidly, and screened in large numbers. However, unlike intestinal samples, environmental field-collected feces may present additional challenges such as matrix variability and environmental degradation due to weathering, UV radiation, and temperature fluctuations.

Analytical evaluation showed high sensitivity and specificity of the new method. For *E. multilocularis*, amplification was consistently achieved down to the 1:256 dilution, corresponding to approximately one egg in 0.10 g of feces (Ct 38.11–38.98), while for *E. granulosus* s.s., the limit of detection was at the 1:512 dilution (Ct 38.32–38.68). Based on these results, a Ct threshold of ≤38.00 was set to identify positive samples for both species. Although absolute Ct values vary across instruments and chemistry, this cutoff aligns with the analytical sensitivity reported in previous copro-qPCR studies detecting low egg loads [55]. The use of 0.10 g of feces was intentionally selected based on the extraction kit protocol and to standardize the assay, while allowing efficient processing and storage of small sample volumes. Specificity testing confirmed no cross-reactivity with bacterial DNA or non-*Echinococcus* taenids, achieving an analytical specificity of 100%, in line with WOAH reference standards [54,56]. Regarding analytical specificity, the exclusivity panel was structured around phylogenetically closely related taeniids and sympatric cestodes circulating in wild and domestic carnivores in north-eastern Italy (*T. krabbei*, *T. polyacantha*, *T. crassiceps*, *T. hydatigena*, *Dipylidium caninum*, and *Hydatigera taeniaeformis*), which represent the primary sources of potential cross-reactivity due to their host overlap and high genomic sequence homology with *Echinococcus*. While *E. coli* was tested as a representative enteric strain, broader coverage of complex intestinal microflora was ensured through the analysis of target-negative fecal matrices alongside comprehensive *in silico* BLAST evaluations. These analyses confirmed that primers and probes do not share target homology with other common gastrointestinal parasites of canids (*Toxocara* spp., *Ancylostoma* spp., *Giardia* spp., *Cryptosporidium* spp.), and no non-specific amplification or probe fluorescence was detected across any tested species or fecal matrix. Moreover, multiplex testing on mixed DNA samples demonstrated reliable simultaneous detection of both species without interference, confirming the assay’s suitability for multiplex use. In fact, no evidence of a masking effect or target competition was observed, even at different concentration ratios, supporting the high specificity and efficiency of the primers and probes used in the multiplex assay. Beyond analytical sensitivity and specificity, the assay demonstrated high intra-laboratory repeatability across testing days and operators (%CV range:0.71%–3.66%), with an overall repeatability of ≥ 95%,consistent with the performance criteria described by WOAH for diagnostic assays [54,56].

The low number of positive field samples (8 *E. multilocularis* and 4 *E. granulosus* s.s. positive samples) and the non-targeted sampling design limit the assessment of diagnostic sensitivity under field conditions. In contrast, the analytical sensitivity of the assay was specifically evaluated through dedicated sensitivity testing. Therefore, the field assessment primarily supports the applicability and feasibility of the assay on naturally collected fecal samples rather than providing a robust estimate of diagnostic sensitivity. Overall, the analytical performance of the multiplex real-time PCR indicates good reproducibility and suitability for large-scale screening.

The assay allows high-throughput screening by enabling simultaneous DNA extraction and amplification of multiple samples, with parasite-specific primers and probes, eliminating the need for DNA sequencing, thereby reducing both the time and cost of analysis. The multiplex real-time PCR method demonstrates several strengths, including effective exclusion of inhibitory substances commonly present in feces (e.g., calcium, tannic acid, humic acid). The use of an internal control (IC) and a DNA extraction kit optimized for fecal material further enhances analytical reliability. Specifically, samples showing PCR inhibition (indicated by the non-amplification of the IC) are diluted 1:10 in nuclease-free water, and if inhibition persists, diluted 1:1 during the DNA extraction phase.

The multiplex real time PCR developed in this study is a qualitative diagnostic tool designed to detect the presence or absence of *E. multilocularis* and *E. granulosus* s.s. DNA. Ct values derived from this assay must be interpreted strictly as qualitative indicators and cannot be used to estimate parasite egg burden in field-collected fecal samples. In natural infections, variables such as uneven egg distribution within the fecal matrix, intermittent shedding, and differential DNA degradation decouple Ct values from the actual number of eggs present. We are aware that the presence of *Echinococcus* DNA in feces may be not exclusively attributable to eggs, but may also result from other parasite material, including worm tissue and, to a lesser extent, larval stages following ingestion of infected intermediate hosts. Nevertheless, eggs were considered the most practical and reproducible biological unit for spiking EENF and obtaining standardized data to support the assessment of analytical sensitivity. A threshold of Ct ≤ 38.00 was applied as a conservative cut-off to prioritize high assay specificity and prevent false positives. We acknowledge that in field samples, biological variability including minor PCR inhibitors or low DNA yields could slightly elevate Ct values, potentially increasing the risk of false negatives. However, building upon the prior optimization and validation by Maksimov et al. [46] and Knapp et al. [52], this shared threshold ensures a reliable, unbiased qualitative performance across both target species.

A primary technical consideration in this work is the asymmetric approach used to establish the analytical Limit of Detection (LoD). While the *E. multilocularis* assay was calibrated using feces spiked with intact, microscopically verified eggs, *E. granulosus* s.s. was evaluated using serial dilutions of extracted fecal DNA. Obtaining biological specimens (adult worms or fresh eggs) of *E. granulosus* s.s. is notoriously difficult compared to *E. multilocularis*; while *E. multilocularis* can be harvested post-mortem from wild definitive hosts (such as red foxes), *E. granulosus* s.s. primarily cycles in dogs, which are alive and routinely subjected to veterinary care and deworming, making intact parasite material scarcely available. While diluting *E. granulosus* s.s. DNA into negative fecal extracts effectively reproduced matrix induced PCR inhibition and equivalent amplification kinetics to justify the shared Ct ≤ 38.00 cut-off for both targets, this setup did not account for initial egg lysis. Consequently, the current *E. granulosus* s.s. LoD cannot be expressed in terms of egg number per gram of feces and is not directly comparable with the *E. multilocularis* LoD established using feces spiked with a known number of intact eggs. Importantly, the performance of the full extraction and diagnostic workflow was subsequently confirmed by successfully testing naturally positive *E. granulosus* s.s. field samples under real matrix conditions. To fully harmonize the analytical baselines, targeted egg-spiking trials for *E. granulosus* s.s. will be conducted as soon as intact biological material becomes available.

To objectively benchmark our protocol against existing biomolecular techniques, a detailed comparison with published multiplex PCR assays is summarized in Table 5. While some multiplex protocols have been developed [43,48,57], these assays lack an IC to monitor PCR inhibition, which is a well-known issue in fecal matrices. Moreover, some of the available assays detect only a single genetic target or require post-amplification processing, such as sequencing, or gel electrophoresis, for target identification [50]. Our assay combines automated fecal DNA extraction, TaqMan specificity, high analytical sensitivity, and an exogenous IC to confirm extraction efficiency and monitor inhibition into a rapid multiplex workflow suitable for high-throughput surveillance.

**Table 5:** Comparison of molecular methods for the detection of *Echinococcus* spp. in definitive hosts. The table summarizes the target species, molecular targets, sample matrices, limits of detection (LoD), use of internal extraction/amplification controls, post-PCR processing requirements, and main methodological advantages and limitations of previously published assays aimed at multiple detection and the assay developed in the present study. Abbreviation: *Em* (*Echinococcus multilocularis)*; *Eg* (*Echinococcus granulosus* s.s.); *Ec* (*Echinococcus canadensis)*; IC (Internal Control), ND (Not Determined), SFCT (Scraping, Filtration and Counting Technique).

Assay	Target species	Target gene(s)	Sample matrix	Analytical LoD	Internal/inhibition control	Post-PCR processing	Main advantages/limitations
**Present study**	*E. multilocularis*, *E. granulosus* s.s.	*rrnL* (Em), *cox1* (Eg)	Canid feces (0.10 g)	1 egg/0.10 g (*Em*); Highest positive DNA dilution (egg count ND) (*Eg*)	Yes	None	Multiplex real-time PCR; simultaneous qualitative detection of both species in a closed-tube assay; internal inhibition control; 100% analytical specificity
**Trachsel et al. [43]**	*E. multilocularis,* *E. granulosus s.l., Taenia spp*	*nad1* (*Em, Eg*); 12S rRNA (*Taenia* spp.)	Carnivore feces; concentrated taeniid eggs	1 egg	No	Agarose gel electrophoresis; sequencing and/or RFLP for species/genotype identification	Multiplex endpoint PCR; detection of *E. multilocularis*, *E. granulosus* and *Taenia* spp.; requires egg concentration and gel electrophoresis; limited species specificity
**Dinkel et al. [44]**	*E. multilocularis* Host species	12S rRNA	Canid feces	10 fg DNA	Yes	None	Simultaneous detection of parasite and host species
**Shang et al.** [57]	*E. granulosus* s.s., *E. multilocularis*, *E. canadensis*	*atp6* (Eg), *nd1* (Em), *rrnL* (Ec)	Parasite lesions/feces	5 pg/µL for each species tested individually; 50 pg/µL (*Eg*), 10 pg/µL (*Em*), and 5 pg/µL (Ec) for mixed species	No	Gel electrophoresis	Detection of three species; endpoint PCR requires gel analysis; no extraction/inhibition control
**Zhang et al. [48]**	*E. multilocularis*, *E. granulosus* s.s., *E. shiquicus*	COI region	Feces/environmental soil samples	10 copies/µL	No	None	Detection of three species; no extraction/inhibition control

Historically, necropsy-based methods such as the Sedimentation and Counting Technique (SCT) or Segmental and Sedimentation Counting Technique (SSCT) have been a standard approach to identify intestinal parasites [37,58], but these require 3–4 hours of intensive work per host, depending on specialized parasitological expertise, and are applicable only to dead animals. Similarly, conventional molecular workflows rely on endpoint PCR assays, such as the widely used copro-PCR developed by Trachsel et al. [43] or the multiplex protocol by Shang et al. [57]. These assays require post-PCR processing (e.g., gel electrophoresis) and substantial hands-on time, often extending total turnaround times to 48–72 hours per batch when coupled with sequencing for target confirmation. In contrast, the real-time multiplex PCR method developed in this study provides high analytical specificity and sensitivity while allowing the processing of up to 96 fecal samples in approximately 3.5 hours from sample preparation to results. By eliminating post-PCR handling, gel electrophoresis, and sequencing, the workflow reduces overall analysis time and reagent consumption, with estimated savings of 40–60%. These features make the assay suitable for large-scale epidemiological screening without requiring specialized parasitological expertise.

Comparable studies, such as Zhang et al. [48], developed multiplex real-time PCR assays for three *Echinococcus* species (*E. multilocularis*, *E. granulosus*, and *E. shiquicus);* however, the method lacked some important features necessary for affordability and reliability. For example, it did not include an IC to assess potential PCR inhibitors (e.g., bile salts and humic acids), which are commonly present in fecal matrices and may cause false-negative results. Additionally, their sensitivity evaluation was expressed in genomic copies, providing a standardized molecular measure of analytical sensitivity, whereas our assay used egg-spiked fecal samples as a biological reference for assessing analytical sensitivity. Independently of this difference, our assay incorporates an exogenous IC with consistent Ct values, alongside a commercial magnetic bead-based extraction kit. This combination provides an additional quality-control measure for monitoring extraction and amplification performance and contributes to the overall robustness and reproducibility of the workflow, as reflected by the low inter-operator variability (%CV range:0.71%–3.66%)

Application of the assay to field samples collected from 250 wild carnivores in 2024–2025 confirmed species-specific patterns of parasite detection. *E. multilocularis* was detected only in red foxes, whereas *E. granulosus* s.s. was detected exclusively in wolves. These findings are consistent with the known ecological behaviours of these parasites and their host preferences. Furthermore, *E. multilocularis* was detected mainly in locations corresponding to previously described foci in the Autonomous Province of Bolzano/Bozen – South Tyrol [50]. On the other hand, the detection of *E. granulosus* s.s. in wolves was confined to the Province of Trento. This finding represents the first detection in this study area using this method for *E. granulosus* s.s. in this host species, although the parasite was previously reported in shepherd dogs and transhumant flocks [27]. These flocks spend the winter periods roaming in the plains of the Veneto and Friuli Venezia Giulia regions, then moving to the mountain pastures during the summer, including ones in Trentino [59], where they may represent a potential source of infection for wolves, during predation. However, since these findings rely on opportunistic samples collected through routine passive surveillance (hunted animals and retrieved carcasses), systematic surveillance is needed for formal epidemiological confirmation.

## 5. Conclusions

The multiplex real-time PCR method developed in this study is a highly sensitive, specific diagnostic tool for the simultaneous detection of *E. multilocularis* and *E. granulosus* s.s. directly from fecal samples of domestic and wild canids. The assay proposed in this study demonstrated 100% analytical specificity and very high sensitivity, reliably detecting as little as a single egg in 0.10 g of fecal material. This approach is particularly suitable as a screening method for surveillance of echinococcosis in domestic and wild carnivores. By facilitating high-throughput analysis and enabling early detection, this assay provides a valuable tool to monitor parasite spread and generate actionable data for public health interventions.

The good performances of the method were demonstrated through its use in the analysis of selected samples from wild canids (i.e., red foxes and wolves) that provided sound and relevant results. A wider use of the method in the future in the whole territory of north-eastern Italy can provide further evidence of its value.

## Supporting information

S1 Table Raw cycle threshold (Ct) values obtained for the assessment of the analytical sensitivity of the real-time PCR assay for *Echinococcus multilocularis* in spiked faecal samples. Four replicates were tested at each target concentration. The logarithm of the number of eggs, together with the mean Ct value and standard deviation (SD), is also reported for each concentration level.
(XLSX)

S2 Table Limits of detection and amplification results for *Echinococcus granulosus* s.s. in dog feces. Results of the real-time PCR amplification of DNA extracted from positive dog fecal samples serially diluted.
(XLSX)

S3 Table Amplification results of mixed *E. multilocularis* and *E. granulosus* s.s. **DNA dilutions tested for Cross-Detection Trials.** FAM: fluorophore relative to *E. multilocularis*; Cy5: fluorophore relative to *E. granulosus* s.s.
(XLSX)

S4 Table Extracted DNA amplified for specificity test and their relative Cycle Threshold (Ct). FAM: fluorophore relative to *E. multilocularis*; Cy5: fluorophore relative to *E. granulosus* s.s.; ID number: identification number of the sample. The symbol “--” indicates that no amplification occurred (Ct value not detected). Sample IDs correspond to institutional IZSVe diagnostic unit codes: IZS = Istituto Zooprofilattico Sperimentale delle Venezie; PAR = parasitology; BL = Belluno; TV = Treviso.
(XLSX)

S5 Table Intra-laboratory repeatability assessment for *E. multilocularis* multiplex real-time PCR. Raw Ct values, mean, standard deviation (SD), and coefficient of variation (%CV) obtained by two operators across three independent testing days and three target contamination levels (High, 1:2; Medium, 1:16; Low, 1:256).
(XLSX)

S6 Table Intra-laboratory repeatability assessment for *E. granulosus* s.s. multiplex real-time PCR. Raw Ct values, mean, standard deviation (SD), and coefficient of variation (%CV) obtained by two operators across three independent testing days and three target contamination levels (High, 1:2; Medium, 1:16; Low, 1:256).
(XLSX)

S7 Table Raw data from wild canid fecal samples analysed for *Echinococcus multilocularis* (Em) and *Echinococcus granulosus* s.s. **(Eg) collected during the 2024–2025 sampling period.** For each sample, the table reports the host species (wolf or red fox), the qualitative PCR results for Em and Eg, the cycle threshold (Ct) value obtained for the target(s), and the Ct value of the internal control (IC). A value of 0 indicates a negative result.
(XLSX)
